# Sialyl Lewis^X/A^ and Cytokeratin Crosstalk in Triple Negative Breast Cancer

**DOI:** 10.3390/cancers15030731

**Published:** 2023-01-25

**Authors:** Carlota Pascoal, Mylène A. Carrascal, Daniela F. Barreira, Rita A. Lourenço, Pedro Granjo, Ana R. Grosso, Paula Borralho, Sofia Braga, Paula A. Videira

**Affiliations:** 1 UCIBIO, Applied Molecular Biosciences Unit, Department of Life Sciences, NOVA School of Science and Technology, Universidade NOVA de Lisboa, 2819-516 Caparica, Portugal; 2 Associate Laboratory i4HB—Institute for Health and Bioeconomy, NOVA School of Science and Technology, Universidade NOVA de Lisboa, 2819-516 Caparica, Portugal; 3 CDG & Allies—Professionals and Patient Associations International Network (CDG & Allies—PPAIN), 2819-516 Caparica, Portugal; 4 Instituto de Anatomia Patológica, Faculdade de Medicina da Universidade de Lisboa, 1649-028 Lisboa, Portugal; 5 Unidade de Mama, Instituto CUF de Oncologia, 1998-018 Lisboa, Portugal; 6 NOVA Medical School, Faculdade de Ciências Médicas, Universidade Nova de Lisboa, 1150-082 Lisbon, Portugal

**Keywords:** triple-negative breast cancer (TNBC), sialyl Lewis^X/A^ (sLe^X/A^), cytokeratin expression, intermediate filament proteins, disease-free survival rate, α6 integrin, aberrant glycosylation

## Abstract

**Simple Summary:**

Triple-negative breast cancer (TNBC) is aggressive, highly metastatic, and associated with poor patient prognosis. Sialyl-Lewis X and A (sLe^X/A^) are sugars with important roles in cell signalling and metastasis. We aimed to describe the relevance of sLe^X/A^ in TNBC patients and its association with other biomarkers. We identified that sLe^X/A^ negatively correlated with cytokeratins, structural proteins present at the cell cytoskeleton, and are involved in cell attachment, by using patient tissues, cell lines, and datasets. Our data suggests that sLe^X/A^ is decorating proteins such as integrin alpha 6, deregulating cell signalling responsible for hemidesmosome formation, impacting cell adhesion, and promoting metastatic behaviour. This work highlights sLe^X/A^ as an important biomarker behind TNBC malignancy to target and treat this breast cancer type.

**Abstract:**

Triple-negative breast cancer (TNBC) encompasses multiple entities and is generally highly aggressive and metastatic. We aimed to determine the clinical and biological relevance of Sialyl-Lewis X and A (sLe^X/A^)—a fucosylated glycan involved in metastasis—in TNBC. Here, we studied tissues from 50 TNBC patients, transcripts from a TNBC dataset from The Cancer Genome Atlas (TCGA) database, and a primary breast cancer cell line. All 50 TNBC tissue samples analysed expressed sLe^X/A^. Patients with high expression of sLe^X/A^ had 3 years less disease-free survival than patients with lower expression. In tissue, sLe^X/A^ negatively correlated with cytokeratins 5/6 (CK5/6, which was corroborated by the inverse correlation between fucosyltransferases and CK5/6 genes. Our observations were confirmed in vitro when inhibition of sLe^X/A^ remarkably increased expression of CK5/6, followed by a decreased proliferation and invasion capacity. Among the reported glycoproteins bearing sLe^X/A^ and based on the STRING tool, α6 integrin showed the highest interaction score with CK5/6. This is the first report on the sLe^X/A^ expression in TNBC, highlighting its association with lower disease-free survival and its inverse crosstalk with CK5/6 with α6 integrin as a mediator. All in all, sLe^X/A^ is critical for TNBC malignancy and a potential prognosis biomarker and therapeutic target.

## 1. Introduction

Breast cancer (BC) is notorious for its heterogeneity, making personalised medicine difficult to implement in clinical settings. Triple-negative breast cancer (TNBC) is one of the most aggressive types of BC, with a poor prognosis, advanced stage, and an increased risk of visceral or brain metastasis [1]. At the time of diagnosis, these tumours are typically larger in size, have a higher histological grade (III) and involve lymph nodes [2]. TNBC is distinguished from other types of BCs by the absence of expression of estrogen receptors (ER), progesterone receptors (PR) and epidermal growth factor receptors type 2 (HER2), representing approximately 15% of all types of BCs [3]. TNBC is mainly treated with chemotherapy because it does not respond to endocrine or anti-HER2 directed therapy and, as such, only a few targeted therapies have been approved [3]. In addition, TNBC’s patients have shorter disease-free survival and overall survival compared to other BC patients. Noteworthy is that more than 70 % of women with metastatic TNBC do not survive more than five years after initial diagnosis [4].

Since TNBC encompasses multiple and heterogeneous entities with unique profiles, attempts to subtype TNBC have been intensively explored to implement biomarker-driven therapeutic approaches [5]. The most studied biomarkers, which include the intermediate filament cytokeratin (CK)5/6, epidermal growth factor receptor (EGFR), P-cadherin and androgen receptor (AR), showed limited clinical value [6,7,8]. Furthermore, it is still unclear which mechanisms underpin TNBC and which factors influence its invasive and metastatic behaviour.

During tumour progression and metastasis, tumour cells initiate the epithelial-mesenchymal transition program, becoming motile cells capable of circulating to other organs [9]. In this process, the interaction of protein complexes called hemidesmosomes (HD) with the microenvironment determines the strength of tumour cells’ adhesion to the extracellular matrix (ECM). These interactions are mediated by its composing HD adhesion molecules including integrin α6β4. When integrins are phosphorylated, the HD disassemble, allowing the intermediate cytoskeleton filaments to detach from the ECM and activate intracellular signalling pathways (e.g., EGFR/PI3K/Akt and FAK/Src), resulting in a more tumorigenic profile [10,11].

While circulating in the bloodstream, tumour cells with the capacity to interact and bind to vascular endothelium can invade distant organs. This is accomplished by the binding of tumour cells to E-selectins expressed by endothelial cells. E-selectins are adhesion molecules required for leukocyte recruitment during the early stages of inflammation [12,13]. They are induced by inflammatory cytokines and constitutively expressed in cancer and marrow microvasculature [12,14]. When they bind to their ligands, they set off a chain of events that leads to the cell adhesion and subsequent transendothelial migration [15,16]. E-selectins bind to sialofucosylated glycans, namely sialyl Lewis X (sLe^X^) and sialyl Lewis A (sLe^A^), displayed on cell surface proteins and acts as their major ligands [17,18]. The protein scaffold modulates the presentation of sLe^X/A^ and subsequent interaction with E-selectin and intracellular signalling [19,20]. The overexpression of the E-selectin ligands (E-SL) is frequently observed in cancer, mostly due to the increased activity of α1,3-fucosyltransferases that catalyse terminal fucosylation steps [21].

There are numerous reports on the elevated sLe^X/A^ expression in BC, namely in tissues, cell lines, and serum [22,23,24]. Interestingly, its expression is higher in metastatic lesions than in primary tumour tissues and, in some cohorts, it correlates with poor prognosis [25,26]. E-SL are also known to be expressed by TNBC [27], yet their relevance and the importance of sLe^X/A^ has not been properly addressed.

In this study, we examined the expression of sLe^X/A^ in TNBC tissue from a cohort of patients. It was observed that sLe^X/A^ was expressed in all TNBC and associated with decreased disease-free survival. We also identified a negative correlation between the immunohistochemical expression of the sLe^X/A^ and CK5/6. Moreover, inhibition of sLe^X/A^ in a BC cell model increased the expression of CK5/6, and reduced cell proliferation and invasion capacity. In our cell model, sLe^X/A^ decorates α6 integrin and its expression impacts the phosphorylation of the downstream intracellular signalling. Collectively, these findings unveil a molecular interplay between sLe^X/A^ and intermediate filaments. It also suggests that this glycan may be exploited to enable better patient stratification and as a therapeutic target in TNBC.

## 2. Materials and Methods

### 2.1. Patients and Clinical Data

This study was performed after institutional ethical approval (Internal Reference Code – SBraga2014) and patient informed consent. It involved 50 TNBC patients who underwent surgery in Hospital CUF Descobertas (Lisboa, Portugal). For each patient, tissue specimens were fixed in formalin and embedded in paraffin. The determination of the molecular subtype was performed by the Hospital’s pathology laboratory. A summary of the clinical data is available in Table S1 of Supplementary Material File S2.

### 2.2. Immunohistochemical Staining of TNBC Sections

Paraffin-embedded sections of TNBC specimens were deparaffinised in trilogy pre-treatment solution (Cell Marque, Rocklin, CA, USA) at 94 °C and antigens recovered in Lab Vision PreTreatment Module (Thermo Scientific, Waltham, MA, USA). After blocking endogenous peroxidase (Atom Scientific, Hyde, UK), sections were stained with anti-CK5/6 (1:200; Cell Marque, Rocklin, CA, USA), anti-AR (1:100; Cell Marque, Rocklin, CA, USA), anti-P-cadherin (1:1000; Abcam, Cambridge, UK) or anti-EGFR (1:100; Abcam) for 1 h in “Diamond: Antibody Diluent” (Cell Marque, Rocklin, CA, USA). We used the previously described immunohistochemical staining protocol for sLe^X/A^ and E-SL detection [27,28]. sLe^X/A^ was stained with the HECA-452 antibody clone (1:50, Biolegend, San Diego, CA, USA), whilst E-SL were stained with E-Immunoglobulin (Ig) chimaera (1:100) followed by anti-Cluster of Differentiation (CD) 62E monoclonal antibody (mAb) (1:50; BD Biosciences, Franklin Lakes, NJ, USA) in Tris-Buffered Saline with Tween, which in case of E-Ig staining was supplemented with 2 mM CaCl2, as previously described [27]. All slides were stained with “HiDef Detection HRP Polymer System” (Cell Marque, Rocklin, CA, USA). The colour was developed using 3,3’-diaminobenzidine solution (ScyTek Laboratories, Logan, UT, USA). Subsequently, sections were stained with hematoxylin (Bio-Optica, Milan, Italy) for nuclear contrast staining before dehydration, clearing, and mounting with Quick-D mounting medium. The slides were visualized under a light microscope with a coupled camera by two certified independent pathologists. The localisation of the expression and the abundance of stained cells was scored using 4 categories: absence or very weak labelling (0); weak staining of less than 1/3 of the cells (1); moderate intensity staining of 2/3 of cells (2), and strong staining of 3/3 of the cells (3).

### 2.3. Cell Culture and Treatments

The human BC cell line, CF1_T, was obtained and immortalised with human telomerase reverse transcriptase cDNA transduction, as described previously [20,28]. It was cultured in Dulbecco’s modified Eagle medium (Sigma-Aldrich, Saint Louis, MO, USA), supplemented with foetal bovine serum, glutamine, penicillin, and streptomycin in T25 flasks at 15% confluence. To reduce the expression of cell surface sLe^X/A^ and E-SL, CF1_T cells were passed, concurrently treated for 5 days with 1 mM 2-fluorofucose (2-FF, Biosynth Carbosynth, Compton, UK) with a change to new 2-FF supplemented medium at the third day, and then analysed.

### 2.4. Fluorescence Microscopy

Cells were cultured on round coverslips inside 24 well plates (Orange Scientific, Braine-l’Alleud, Belgium) for 24 h. Then cells were washed, fixed, and permeabilised with 0.1% TritonX100 and blocked with 1% bovine serum albumin (BSA) (Sigma-Aldrich, Saint Louis, MO, USA). Cells were subjected to immunofluorescence staining with anti-sLe^X/A^ (1:50; HECA-452 clone; Biolegend, San Diego, CA, USA) and anti-CK5/6 (1:200; Cell Marque, Rocklin, CA, USA). Cells were incubated with anti-rat IgM-Fluorescein isothiocyanate (FITC) (1:50) (BD Pharmingen, San Diego, CA, USA) or anti-mouse IgG-FITC (1:100) (Dako, Santa Clara, CA, USA) secondary antibodies. Finally, nuclei were stained with 4′,6-diamidino-2-phenylindole (1μg/mL, ThermoFisher, Waltham, MA, USA) for 10 min, washed, mounted with montage medium Mowiol+1,4-diazabicyclo [2.2.2]octan, and analysed. Fluorescence intensities from five randomly selected microscopic fields of cells were quantitatively analysed with ImageJ software and using the corrected total cell fluorescence (CTCF) formula: CTCF = Integrated density – (Area of selected cell x Mean fluorescence of background readings).

### 2.5. Western Blot

Cells were lysed using Pierce^®^ Immunoprecipitation Lysis buffer (ThermoFisher, Waltham, MA, USA) supplemented with cOmplete™, Mini, ethylenediaminetetraacetic acid-free Protease Inhibitor Cocktail (Roche, Basileia, Switzerland). The protein was quantified with the Pierce^TM^ bicinchoninic acid Protein Assay kit (ThermoFisher, Waltham, MA, USA) following the manufacturer’s instructions. An 8% acrylamide sodium dodecyl sulfate–polyacrylamide gel electrophoresis gel was loaded with 30 μg of denatured protein. After the run, proteins were transferred to a nitrocellulose membrane (Amersham^TM^ Protran^®^ Premium 0.45 μm NC, Amersham, UK) and blocked with Carbo-Free^TM^ Blocking solution (VectorLabs, Newark, CA, USA). Incubation with a mouse anti-human plectin IgG1 primary antibody (SantaCruz Biotechnology, Dallas, TX, USA) or mouse monoclonal anti-α-tubulin antibody (Sigma-Aldrich, Saint Louis, MO, USA) was performed overnight at 4 °C. Secondary detection with the goat anti-mouse horseradish peroxidase antibody was performed for 1 h at room temperature (BD Biosciences, Franklin Lakes, NJ, USA). Signal was revealed using the Lumi-Light Western Blotting Substrate (Roche, Basileia, Switzerlandand X-ray films (Amersham Hyperfilm^TM^ ECL, Amersham, UK). Membrane stripping was achieved using the Restore^TM^ Western Blot Stripping Buffer (ThermoFisher, Waltham, MA, USA).

The western blot bands quantification was conducted using the ImageJ software v.1.53 (File S1).

### 2.6. Flow Cytometry

To analyse the expression of intracellular markers by flow cytometry, cells were first permeabilised using Fixation/Permeabilization Solution Kit (BD Biosciences, Franklin Lakes, NJ, USA). Cells were stained with 1:1000 dilution of anti-phosphorylated (p)-Src (Tyr416), anti-total Src, anti-p-AKT (Ser473), anti-AKT1/2, anti-p-Erk1/2, anti- Erk1/2, anti-p-p38 MAPK and p38 MAPK mAbs, all from Cell Signalling Technology at 4 °C for 30 min. For cell surface α6 integrin staining, the anti-CD49f antibody was used (BD Biosciences, Franklin Lakes, NJ, USA). A FITC-conjugated anti-mouse IgG (Dako, Santa Clara, CA, USA) was used for secondary detection at 4 °C for 20 min. Cells were washed and fixed with 2% paraformaldehyde. Data were acquired using the Attune Acoustic Focusing Cytometer (Applied Biosystems, Waltham, MA, USA) and analysed with FlowJo software version 10.0.5 (BD Biosciences, Franklin Lakes, NJ, USA). Data were presented as delta mean fluorescent intensity (MFI) obtained by subtracting the MFI of the secondary staining control.

### 2.7. TCGA Analysis

The data from TNBC patients, namely clinical metadata (e.g., patient, tumour stage, follow-up information) as well as RNA-Seq read counts in the form of HTSeq count tables were obtained from various breast invasive carcinoma cases using the ‘brca.data’ R package and pipeline previously described by Lopes et al. [29]. Briefly, clinical data on ER, PR, and HER2 expression were used to select TNBC cases. TNBC patients were considered when all three markers were “negative”. A final dataset of TNBC patients with logarithmically transformed (base 2) transcripts per million was created to access gene expression.

TNBC samples were divided by their high or low gene expression for each of the fucosyltransferases (FUT) gene expressions (*FUT3, 4, 5, 6, 7* and *10*) based on the median of all samples. The expression of the genes that code for CK5/6 (*KRT5, KRT6A, -6B, -6C*) was compared between the high and low FUT expression groups (File S3).

### 2.8. Statistical Analysis

The R v 4.1.1 computational language [30] was used for the following statistical analyses and the scripts made available as Script S1–S3. The disease-free survival (DFS) was estimated using the Kaplan–Meier approach and compared between groups using the log-rank test employing the survival (v3.4-0) [31] and the survminer (v0.4.9) R packages. We used the Shapiro–Wilk normality test to determine the normality of variables. The non-parametric Spearman correlation was used to examine the relationship between the data. Using the GraphPad Prism 8.4 (GraphPad Software, Inc., San Diego, CA, USA), the unpaired *t*-tests were performed to compare E-SL/sLe^X/A^ expression between the high and low CK5/6 expression groups and cell signalling proteins’ expression between 2-FF treated and non-treated cells. An unpaired Student’s t-test with Welch’s correction was used to compare CK genes expression between the high and low FUTs expression. Tests were considered statistically significant when *p* < 0.05 (*), *p* < 0.01 (**), *p* < 0.001 (***), *p* < 0.0001 (****) or marginally significant when 0.05 < *p* < 0.1. All bar plots were created using GraphPad Prism 8.4.

## 3. Results

### 3.1. Biomarker Characterisation and Correlation with Clinical Features

**Figure 1:**
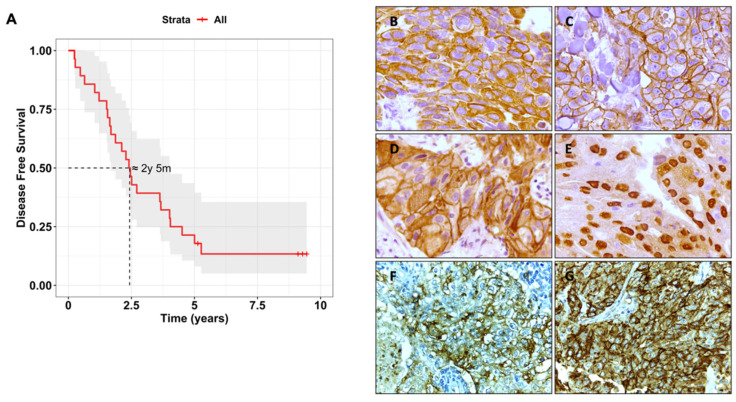
Disease-free survival (DFS) of the triple-negative breast cancer (TNBC) patient cohort and respective immunohistochemical biomarker staining of respective TNBC tissues. (**A**) Kaplan–Meier curve of DFS of all TNBC patients enrolled in this study generated with the survminer (v0.4.9) and survival (v3.4-0) R packages. Representative microphotographs (400X magnification) of immunohistochemical staining against cytokeratin (CK) 5/6 (**B**), P-cadherin (**C**), Epidermal Growth Factor Receptor (EGFR) (**D**), Androgen Receptor (AR) (**E**), Sialyl-Lewis X and A (sLe^X/A^) (**F**) and E-selectin ligand (E-SL) (**G**) in TNBC tissue sections. Tissues were stained with hematoxylin, which colours nuclei. The staining with antibodies or E-Ig was followed by a horseradish peroxidase conjugated secondary antibody and visualised in brown.

TNBCs are generally characterised by poor prognosis and heterogeneous biology. In this study, the median disease-free survival (DFS) of our TNBC patient cohort was 802.5 days (Min = 89 days, Max = 1826 days) with a 50% probability of recurrence after approximately 2 years and 5 months (Figure 1A). Most tumours were high grade (56% grade III and 2% grade IV) and poorly differentiated, whilst 26% were of moderate grade (grade II) and 16% were low grade (grade I). The median patient’s age at diagnosis was 64 years (Min = 33 years; Max = 89 years). The median tumour size was 15 mm (Min = 2 mm; Max = 70 mm) and the median number of invaded sentinel lymph nodes by tumour cells was 12 (Min = 1; Max = 25). The clinicopathologic characteristics of this TNBC cohort is shown in Table S1.

To characterise the molecular profile of the TNBC patient cohort, the expression of the biomarkers CK5/6, EGFR, P-cadherin, AR, sLe^X/A^, and E-SL were measured in tumour sections by immunohistochemistry (Figure 1, Table 1). Staining of CK5/6 expression was observed in the cytoplasm (Figure 1B). Its expression was observed weakly in 24% of the cases, moderately in 12% and strongly in 8% of the cases. P-cadherin staining was observed mostly in the membrane in 96% of the cases (Figure 1C, Table 1). EGFR was detected in the cell membrane very strongly in 14% of the cases, while 60% of cases had weak or very weak staining, and 26% had moderate staining (Figure 1D, Table 1). AR staining was mostly nuclear and positive in 34% of the cases (Figure 1E, Table 1). sLe^X/A^ was expressed in all cases, with 30% being strongly expressed while moderately and weakly expressed in 30 and 40% of the cases, respectively. sLe^X/A^ staining was identified mostly both in the cell membrane and cytoplasm (65%), while 15% of the cases presented only cytoplasmic staining, and 20% only cell membrane staining (Figure 1F, Table 1). E-SL was also expressed in all cases, being strongly expressed in 55% of the cases, while weakly expressed in 20% and moderately expressed in 25% of the cases. In 60% of the cases, E-SL staining was found in cytoplasm and plasma membranes. In 25% of the cases, E-SL presented just cytoplasmic staining, while 15% had only on-cell membrane staining (Figure 1G, Table 1).

### 3.2. sLe^X/A^ and E-SL Expression Negatively Correlate with CK5/6 Expression in TNBC

**Figure 2:**
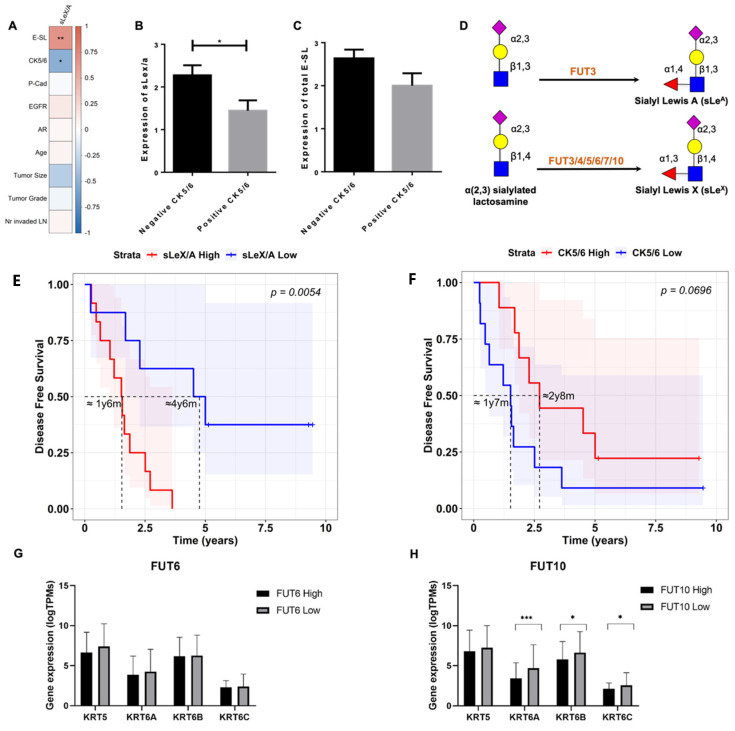
sLeX/A/E-SL and CK5/6 expression in TNBC tissues are inversely correlated and influence DFS. (**A**) sLe^X/A^ is positively correlated with E-SL staining (*p* = 0.002) and negatively correlated with CK5/6 expression (*p* = 0.019) in TNBC tissues. Non-parametric Spearman correlation was used to evaluate the association between features. CK5/6-positive TNBC have lower expression of sLe^X/A^ (**B**) and E-SL (**C**) than the TNBC samples that do not express the CK5/6. TNBC cases were divided in two groups according to CK5/6 expression (score ≥ 1) or lack of expression (score = 0); (**D**) sLe^X^ and sLe^A^ structure and terminal step of addition of fucose by fucosyltransferases (FUT). (**E**) Patients with lower sLe^X/A^ expression have a better 10 year disease-free survival than those with higher expression (*p* = 0.0054); (**F**) Contrastingly, patients with lower CK5/6 expression have worse 10 year DFS than those with higher expression (*p* = 0.0696); Kaplan–Meier curves show the DFS of patients with high (red) and low (blue) sLe^X/A^ or CK5/6 expression (higher and lower expression values than the mean sLe^X/A^/CK5/6 expression, respectively) generated with the survminer (v0.4-9) and survival (v3.4-0) R packages. (**G**) *FUT6* low gene expression group has increased *KRT6A* gene expression (*p* = 0.074)**,** and (**H**) *FUT10* low gene expression group has increased *KRT6A* (*p* = 0.002), *-6B* (*p* = 0.034) and *-6C* (*p* = 0.025), compared with the high expression groups. TNBC tissue genetic data from the TCGA database was analysed for gene expression. Samples were subdivided based on the median expression of *FUT6* and *FUT10* and correlated with genes involved in CK5/6 epitope expression (*KRT5, KRT6A, -6B, -6C*). Legend: * - p < 0.05, ** - p < 0.01, *** - p < 0.001.

We then assessed for correlations between sLe^X/A^ and E-SL for clinicopathologic features and molecular profile. sLe^X/A^ and E-SL reactivity showed a significant and positive correlation (r = 0.660, *p* = 0.002, Figure 2A). Comparing the expression of these two markers with the expression of the other analysed molecules, we verified that sLe^X/A^ expression was negatively correlated with CK5/6 expression (r = −0.516; *p* = 0.019). Similarly, total E-SL expression also tended to be negatively correlated with the expression of CK5/6 (r = −0.443; *p* = 0.051). Comparing the expression of sLe^X/A^ and total E-SL between the negative (expression = 0) and positive (expression ≥ 1) CK5/6 cases, we verified that both markers are significantly more expressed in negative CK5/6 cases compared to the positive ones (Figure 2B,C).

Interestingly, regarding the influence of sLe^X/A^ expression, we found that patients with low expression of sLe^X/A^ had significantly higher DFS (*p* = 0.005) and only reached a 50% probability of disease recurrence after 4 years and 6 months when comparing with patients with high expression of sLe^X/A^ that reached the same probability after 1 year and 6 months (Figure 2E). Further corroborating the negative correlation between sLe^X/A^ and CK5/6, high expression of CK5/6 shows a tendency to higher DFS (marginally significant, *p* = 0.0696), with a 50% DFS probability at approximately 2 years and 8 months, compared to a 1 year and 7 months in the CK5/6 low expression group (Figure 2F). This relationship with the DFS is almost the inverse of what it is seen for the sLe^X/A^ (Figure 2E), reinforcing the negative correlation between these two biomarkers. To further corroborate the inverse correlation between sLe^X/A^/ E-SL and CK5/6 expression, we analysed the gene expression of potential key players in this interaction from 160 TNBC patients publicly available in TCGA. The data retrieved concerned the gene expression of the fucosyltransferases (enzymes responsible for completing the sLe^X/A^ structure during their biosynthesis [32], Figure 2D), namely *FUT3, 4, 5, 6, 7* and *10*, and of the genes responsible for the synthesis of the CK5/6 biomarker epitope, namely *KRT 5, 6A, 6B, 6C*. Supporting our previous observations, we noted that when subdividing TNBC patients into a high or low expression of the fucosyltransferase *FUT6* and of *FUT10*, the groups with lower expression of *FUT6/10* have higher expression of CK6 genes (Figure 2G,H and Figure S1).

Altogether, the data suggest that in TNBC tissues, the higher phenotypic expression of sLe^X/A^ leads to a CK5/6 decrease and vice versa. The data also suggests that the expression of the genes underlying the sLe^X/A^ and CK5/6 biosynthesis are controlled conversely.

### 3.3. sLe^X/A^ Inhibition Leads to Increased CK5/6 Expression in a Breast Cancer Cell Line

**Figure 3:**
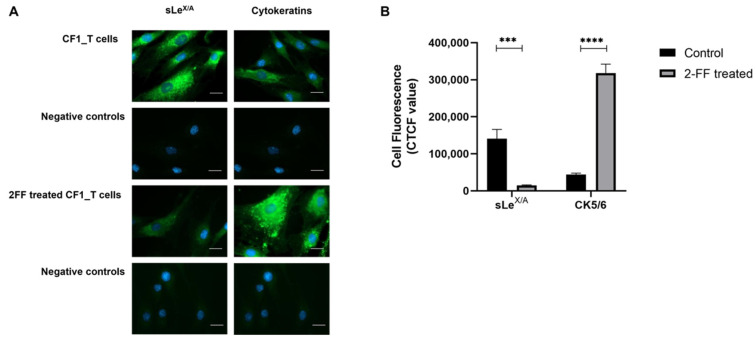
Inhibition of fucosylation decreases sLe^X/A^ expression and increases cytokeratin. CF1_T cells were treated or not with 2-fluorofucose (2-FF) and then labelled with HECA-452 mAb and anti-CK monoclonal antibody (mAb), as described in the materials and methods. (**A**) Fluorescence microscopy images (scale bar: 1 μm). The resulting fluorescence of labelling with HECA-452 and anti-CK is shown per column. The first row presents images of untreated cells stained with primary antibodies; on the second row there are the labelling controls in the absence of primary antibodies; the third row shows CF1_T cells treated with 2-FF and stained with primary antibody, and the fourth row exhibits labelling controls of 2-FF treated cells in the absence of primary antibodies. Images are representative of merging fluorescence where antibody labelling is shown in green, and nuclei were stained with 4′,6-diamidino-2-phenylindole (blue). (**B**) Corrected total cell fluorescence. The graph shows the calculated arbitrary values of corrected total cell fluorescence (CTCF) retrieved from images depicted in A as described in the materials and methods section. Legend: *** - p < 0.001, **** - p < 0.0001.

To further understand the correlation between sLe^X/A^ and CK5/6, we then analysed a BC cell line known to express high levels of sLe^X/A^/E-SL, the CF1_T [28]. After submitting the cells to 2-FF, an inhibitor of fucosylation, sLe^X/A^ expression was reduced compared to untreated cells, as expected (Figure 3A). This is consistent with previous studies, where submitting the cells to the same inhibitor concentration (1 mM) abrogated the cell adhesion to E-selectin due to the absence of its ligands (Figure S2). In addition, cytokeratin expression increased 8-fold after treatment (Figure 3B). While CK5/6 was associated with poor cancer prognosis by some studies [33,34], others have shown that CK5/6 downregulation is associated with increased malignancy of cancer cells [35].

To assess the malignant profile of 2-FF treated cells, which showed increased CK5/6 expression, we set out to study proliferation and invasion in 2-FF treated CF1-T cells. Concordantly with previous reports, the CF1-T cells showed reduced proliferation capacity (Figure S3) and migratory ability (Figure S4).

These findings point to a potential mechanism in which sLe^X/A^ and CK5/6 expressions are inversely regulated. It also suggests that patients with lower expression of sLe^X/A^ have reduced TNBC malignant features.

### 3.4. sLe^X/A^ Decorates α6 Integrin and Affects the Associated Signalling Pathways

Since sLe^X/A^ glycan can decorate different cell surface proteins, we considered that the crosstalk between sLe^X/A^ and cytokeratin could be attributed to changes in intracellular signalling coordinated by a sLe^X/A^-decorated protein. This is consistent with our previous observation that the cellular signal transduction pathways are affected upon inhibition of the sLe^X/A^ biosynthesis [28]. To investigate this, we first interrogated which signalling pathways are affected when cells have lower sLe^X/A^ levels due to 2-FF treatment.

**Figure 4:**
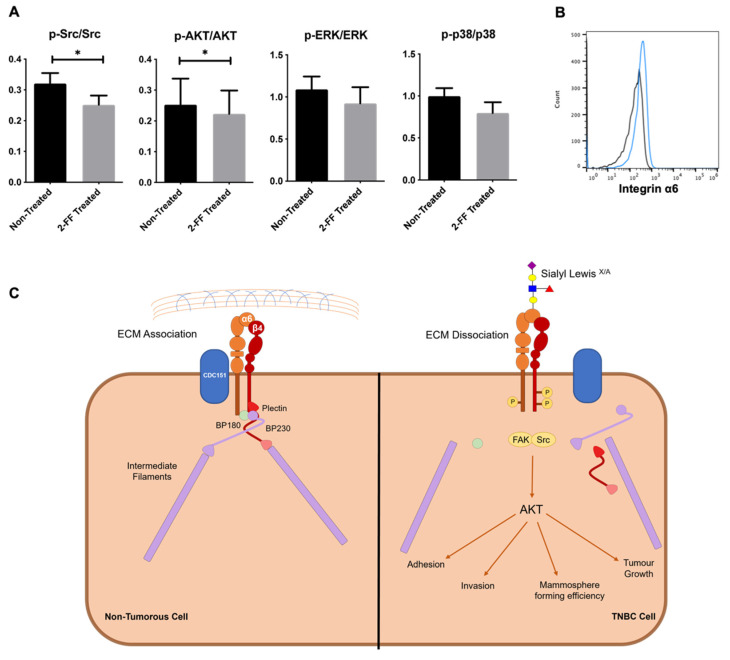
α6 integrin-associated pathways are affected upon 2-FF treatment. (**A**) Expression of phosphorylated proteins is affected by 2-FF. CF1_T cell line treated with 2-FF for 5 days were analysed regarding their expression of phosphorylated Src (p-Src), AKT (p-AKT), ERK1/2 (p-ERK), and respective total proteins by flow cytometry. Ratio between phosphorylated versus total protein expression is represented in the graphs (n = 3, *p* < 0.05 (*)). (**B**) CF1_T cells express α6 integrin. Flow cytometry analysis of CF1_T cells stained with anti-α6 integrin antibody, plus fluorescent secondary antibody. Representative histogram showing positive staining (blue line). Cells stained only with fluorescent secondary antibody and without anti-α6 integrin antibody were used as a negative control (dark line); (**C**) Proposed mechanism of the influence of sLe^X/A^ decoration of α6integrin on cytokeratin expression. The crosstalk between α6 integrin and cytokeratins is established by the hemidesmosome (complex of α6β4 integrin, plectin, BP180, CD151, BP230). sLe^X/A^ decoration activates integrin and the downstream signalling pathways, contributing to a more aggressive phenotype.

The ratio of phosphorylated Src, AKT, ERK1/2 and p38 versus corresponding total protein was assessed by flow cytometry upon 5 days of 2-FF treatment. As shown in Figure 4A, the ratio of the expression of phospho-Src/Src and phospho-AKT/AKT showed a significant reduction in 2-FF treated cells. This suggests that Src and AKT pathways are negatively affected when sLe^X/A^ levels are reduced.

Additionally, we revised which glycoproteins are decorated with sLe^X/A^ (previously reported [20]). Among them, the α6 integrin was identified as a potential player in the link between sLe^X/A^ and cytokeratin with the highest association combined score (Table S2). In addition, the above identified affected Src and AKT pathways are known to be intermediated by α6 integrin [36]. Since the role of α6 integrin is well established in the formation of the HD, particularly complexed with β4 integrin [37], we hypothesise that the signalling mechanisms necessary for the good functioning of the HD might be also impaired.

Flow cytometric analysis confirmed the expression of α6 integrin on the cell surface of CF1-T cells (Figure 4B). We also confirmed the expression of plectin by these cells, a protein reported to be virtually expressed in all mammalian cells and tissues (Figure S5). This agrees with the fact that plectin links the cytoplasmic tail of integrin subunit β4 (complexed to α6 integrin) to cytokeratins. Overall, these data further corroborate that the decoration of α6 integrin with sLe^X/A^ contributes to the deregulation of signalling pathways in the interplay between integrin and cytokeratins linked by plectin (Figure 4C).

## 4. Discussion

Among the subtypes of BC, TNBC is well known for its heterogeneity and aggressiveness, as well as for its patients having the highest mortality rates. According to our findings, TNBC patients had a DFS of 2–3 years, which is consistent with previous reports of a typical poor prognosis [4,38]. The highly variable response between each patient with TNBC prevents patients from being uniformly treated, urging the need for a better understanding of the underlying mechanism for better subtyping that helps prognosis and selection of appropriate therapy for these patients.

In cancer, sLe^X/A^ antigens play an important role in cell migration and metastasis by facilitating extravasation acting as selectin ligands and participating in tumorigenic signalling [20,28,39,40,41]. Despite its well-known association with metastatic lesions in BCe [17,25], its significance in TNBC is still unknown. Therefore, to the best of our knowledge, there is a need to further investigate the potential role of sLe^X/A^ in TNBC metastasis. In the current study, all tested TNBC samples were stained for sLe^X/A^ and E-SL in addition to at least one basal marker, CK5/6, EGFR, AR or P-cadherin, as previously described [42,43]. Our data showed a strong correlation between sLe^X/A^ and E-SL reactivity, suggesting that sLe^X/A^ glycans are the major ligands of E-selectin in TNBC [27].

When comparing the sLe^X/A^ results to the other markers studied, we encountered a negative correlation with CK5/6, which is an important intermediate filament involved in cell adhesion and migration [44]. Interestingly, higher sLe^X/A^ expression and lower CK5/6 expression leads to a faster disease recurrence when looking at their DFS. In silico analysis of the gene expression of CK5/6 regarding the gene expression of the α1,3-fucosyltransferases that catalyse the terminal fucosylation steps (FUT3, FUT4, FUT5, FUT6, FUT7, and FUT10) (Figure 2D) [18,21] revealed a negative association of *FUT6* and *FUT10* with the CK5/6 genes. These findings help to further corroborate a possible antagonistic interaction between the sLe^X/A^ biosynthetic pathway and the CK5/6 pathway.

Since sLe^X/A^ protein scaffolds are critical for cell signalling, it is likely that these proteins strongly influence the CK5/6 expression. Our group previously reported which glycoproteins are decorated with sLe^X/A^ in BC [20]. Using the STRING database [45] of reported protein–protein interactions and computational prediction, we identified that α6 integrin has the highest interaction score with CK5/6 protein. In cancer, α6 integrin is often associated with metastatic behaviour, increasing cancer cells’ migration and invasion, due to the disassembly of the HD [46]. Concordantly, other studies also report that altered glycosylation promotes the phosphorylation of the subunit β4 of integrin and metastasis [47].

To understand the distinctions between the presence and absence of sLe^X/A^, CF1_T cell line was treated with 2-FF, a small molecule fucosyltransferase inhibitor, which inhibits sLe^X/A^ expression. Upon treatment, an increase of CK5/6 expression was observed which further supports prior data regarding a potential opposing interplay between CK5/6 and sLe^X/A^. Treated CF1_T cells also displayed a reduced cancer cell migration and proliferation, which is consistent with the sLe^X/A^ association with a lower DFS. We identified differences between treated and untreated cell lines suggesting the potential involvement of the Src pathway which is a major player in BC cell proliferation and migration [48]. Src acts as a signalling cue for other pathways such as AKT, which was also altered after 2-FF treatment, and EGFR, which was expressed in 70% of the examined patients’ tissue. This information, together with the observation that α6 integrin has the highest CK5/6 interaction score, might indicate that sLe^X/A^ expression in α6 integrin activates the Src pathway. The activation of this pathway could result in: (i) a decrease of the cytokeratin, as cancer cells undergo epithelial-to-mesenchymal transition necessary for a metastatic profile; and (ii) phosphorylation of α6β4 integrin, further facilitated by the decrease of cytokeratin to promote the dissociation of HD. Altogether, these interactions contribute to the migratory and more malignant behaviour of higher expression of sLe^X/A^ TNBC cells.

We recognize some limitations in this study that could be addressed in future research. First, given that this study was achieved through a direct collaboration with the Breast unit of the CUF Oncology Institute (which works exclusively with patients diagnosed with BC), it was not possible to obtain normal samples from apparently healthy individuals. Future studies should include normal samples or, alternatively, peritumoral tissue from the TNBC cancer patients as controls. Second, efforts should be made to validate our observations in a bigger patient cohort. Not only can this increase the strength of the observed correlation but also might allow patient stratification according to sLe^X/A^ levels or other biomarkers. Nevertheless, our analysis was complemented with gene expression data of 160 cases of TNBC retrieved from the TCGA database which reinforced in part the limitation of our patient cohort. It is also important to discuss that we used the CF1_T cell line as one of three models in our study. This cell line, originally obtained from a patient with invasive ductal carcinoma, was selected as a model due to its very low expression of CK5/6 and high expression of sLe^X/A^ [28]. The expression of ER, PR, and HER2 biomarkers is also low. When treated with 2-FF and concurrently with a reduction in sLe^X/A^, there is an increase in CK5/6 expression analysed by microscopic fluorescence, similarly to what is seen when there is low sLe^X/A^ expression in TNBC tissues. The use of this cell line to complement the information derived from TNBC tissues and described in the TCGA dataset supports the inverse crosstalk between sLe^X/A^ and CK5/6 and suggests that our findings may be transversal to other BC types. Finally, other models and lines of research could be used or developed to continue deciphering the reported associations and proposed pathway. Therefore, besides that our results must be confirmed in other in vitro/in vivo TNBC models and in a bigger TNBC patient cohort, this study opens new research avenues to study these mechanisms in TNBC and other BC types. These findings contribute to highlight malignant features to be explored as potential therapeutic targets.

## 5. Conclusions

Our findings contribute to new understanding of TNBC cells adhesion and metastatic behaviour, highlighting a previously unknown crosstalk between sLe^X/A^ and cytokeratin, intermediated by the α6 integrin. Further studies are necessary to clarify the interaction and the role of the proteins involved in this process. Our contribution supports sLe^X/A^ as a critical player in TNBC malignancy and suggests it as a biomarker to target and treat this BC type.

## Figures and Tables

**Table 1:** Distribution of the expression of the biomarkers in the TNBC population tested in this study.

Biomarker	Biomarker Staining ^1^	% of Cases
CK5/6	0	56%
	1	24%
	2	12%
	3	8%
EGFR	0	30%
	1	30%
	2	26%
	3	14%
AR	Negative	66%
	Positive	34%
P-cadherin	Negative	4%
	Positive	96%
sLe^X/A^	0	0%
	1	40%
	2	30%
	3	30%
E-SL	0	0%
	1	20%
	2	25%
	3	55%

^1^ The staining score consists in 0—absence or weak labelling; 1—weak staining of less than 1/3 of the cells; 2—moderate intensity staining of 2/3 of cells: and 3—strong staining of 3/3 of the cells.

## Data Availability

Data is contained within the article or Supplementary Material(s).

## Supplementary Materials

The following supporting information can be downloaded at: https://www.mdpi.com/article/10.3390/cancers15030731/s1, File S1: The Whole Western blot. File S2: Table S1: Patients’ and tumours’ characteristics of the TNBC cohort of this study; Table S2: List of retrieved sLeX/A proteins immunoprecipitated from the CF1_T breast cancer cell line based on a label-free quantification of glycoproteins by mass spectrometry (as reported by Carrascal et al. 2018 and their reported association with Cytokeratin 5/6; Figure S1: CK5/6 genes expression according to the high or low expression of FUTs; Figure S2: CF1_T cells treated with 2-FF lose the ability to bind E-selectin under flow conditions; Figure S3: Proliferation index of CF1_T cells treated with 2-fluorofucose (2-FF); Figure S4: Migratory ability of CF1_T cell line after 2-fluorofucose (2-FF) treatment analysed by scratch wound healing assay; Figure S5: CF1_T cell line expresses plectin. File S3: Script S1: R script with the survival analysis of TNBC patients. Script S2: R script with the correlation analysis of sLeX/A with the other biomarkers and clinical features. Script S3: R script to obtain Cytokeratin epitopes genes expression according to the high and low expression of fucosyltransferases genes. File S4: TNBC patient cohort clinical data.

## Abbreviations

2-FF—2-fluorofucose; AR—Androgen receptor; BC—Breast cancer; CD—Cluster of differentiation; CK5/6—cytokeratins 5/6; CTCF—Corrected total cell fluorescence; DFS—Disease-free survival; E-SL—E-selectin ligands; ECM—Extracellular matrix; EGFR—Epidermal growth factor receptor; ER — Estrogen receptors; FITC—Fluorescein isothiocyanate; FUT—fucosyltransferases; HD—Hemidesmosomes; HER2—Epidermal growth factor receptors type 2; PR—Progesterone receptors; sLe^A^—Sialyl-Lewis A; sLe^X^—Sialyl-Lewis X; TCGA—The Cancer Genome Atlas; TNBC—Triple-negative breast cancer.
