# Supplementary material for: Sialyl LewisX/A and Cytokeratin Crosstalk in Triple Negative Breast Cancer
Source: Cancers (Basel). 2023 Jan 25;15(3):731. doi: 10.3390/cancers15030731 (PMC9913872; doi:10.3390/cancers15030731)
Supplement: Supplementary file 1 [file cancers-15-00731-s001.zip › Supplementary File S1.pdf]

Supplementary File S1

A **B**

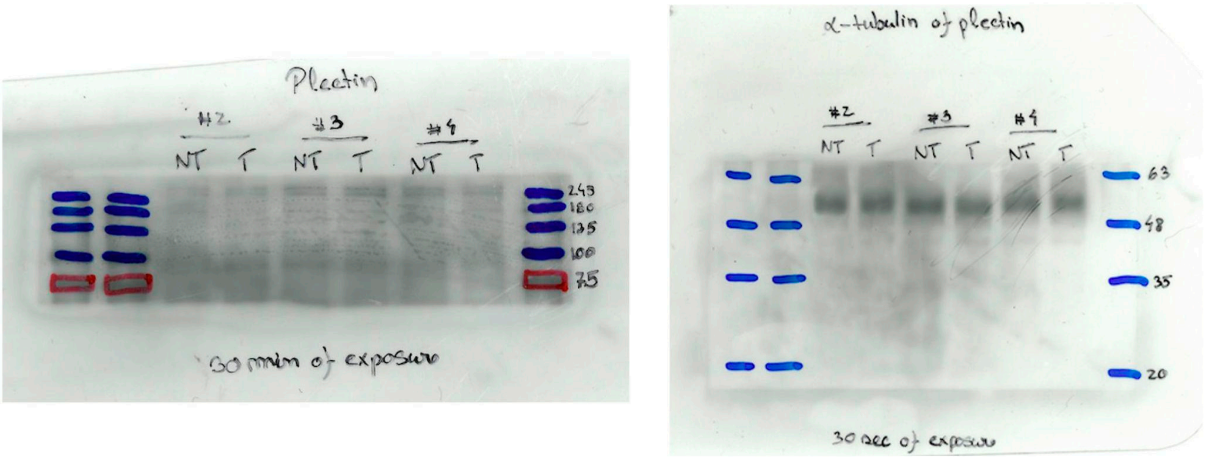

Whole Western blot films showing all bands and molecular weight markers of (A) plectin staining and (B) α-tubulin staining. Lanes #2, #3 and #4 correspond to n=1, n=2 and n=3, respectively. Legend: NT - Not treated; T - Treated (with 2-FF).
