# Supplementary material for: Sialyl LewisX/A and Cytokeratin Crosstalk in Triple Negative Breast Cancer
Source: Cancers (Basel). 2023 Jan 25;15(3):731. doi: 10.3390/cancers15030731 (PMC9913872; doi:10.3390/cancers15030731)
Supplement: Supplementary file 1 [file cancers-15-00731-s001.zip › Supplementary File S2.pdf]

Supplementary File S1

Table S1. Patients' and tumours' characteristics of the TNBC cohort of this study.

TNBC (n = 50)	
**Age at diagnosis (years)**	
Median	64
Min	33
Max	89
**Grade (% of cases)**	
I	16%
II	26%
III	56%
IV	2%
**Tumour size (mm)**	
Median	15
Min	2
Max	70
**# of invaded lymph nodes**	
Median	12
Min	1
Max	25

Table S2. List of retrieved $sLe^{X/A}$ proteins immunoprecipitated from the CF1_T breast cancer cell line based on a label-free quantification of glycoproteins by mass spectrometry (as reported by Carrascal et al 2018 and their reported association with Cytokeratin 5/6. The association of each protein with cytokeratin 5/6 genes is annotated according to the STRING database for protein-protein interactions (Szklarczyk et al. Nucleic acids research 47.D1 (2018): D607-D613.2). When there is a positive association, STRING combined scores are annotated. *The combined score refers to the value of interaction between Cytokeratin 5 and $sLe^{X/A}$ proteins. It accounts for co-expression, experimental/biochemical data, association in curated databases and co-mention in PubMed abstracts. This interaction is highlighted in the picture by a binding string. Proteins are presented by highest to lower combined scores.

Entry	Entry name	Protein names	Positive association with cytokeratin	Combined Score*
P23229	ITA6_HUMAN	Integrin alpha-6 (CD49 antigen-like family member F) (VLA-6) (CD antigen CD49f) [Cleaved into: Integrin alpha-6 heavy chain; Integrin alpha-6 light chain; Processed integrin alpha-6 (Alpha6p)]	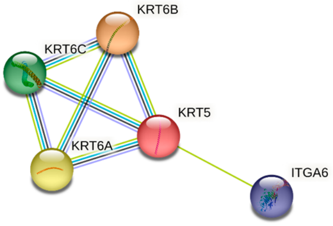	0.658
P16070	CD44_HUMAN	CD44 antigen (CDw44) (Epican) (Extracellular matrix receptor III) (ECMR-III) (GP90 lymphocyte homing/adhesion receptor) (HUTCH-I) (Heparan sulfate proteoglycan) (Hermes antigen) (Hyaluronate receptor) (Phagocytic glycoprotein 1) (PGP-1) (Phagocytic glycoprotein I) (PGP-I) (CD antigen CD44)	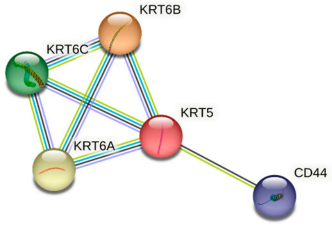	0.616
O60716	CTNND1_HUMAN	Catenin delta-1 (Cadherin-associated Src substrate) (CAS) (p120 catenin) (p120(ctn)) (p120(cas))	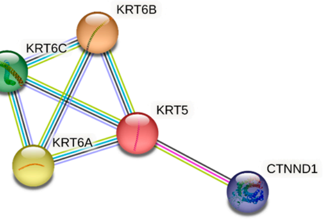	0.554
P35613	BASI_HUMAN	Basigin (5F7) (Collagenase stimulatory factor) (Extracellular matrix metalloproteinase inducer) (EMMPRIN) (Leukocyte activation antigen M6) (OK blood group antigen) (Tumor cell-derived collagenase stimulatory factor) (TCSF) (CD antigen CD147)	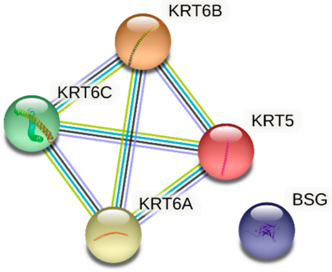	N/A
P15144	AMPN_HUMAN	Aminopeptidase N (AP-N) (hAPN) (EC 3.4.11.2) (Alanyl aminopeptidase) (Aminopeptidase M) (AP-M) (Microsomal aminopeptidase) (Myeloid plasma membrane glycoprotein CD13) (gp150) (CD antigen CD13)	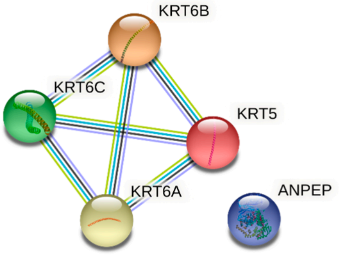	N/A
P05556	ITB1_HUMAN	Integrin beta-1 (Fibronectin receptor subunit beta) (Glycoprotein IIa) (GPIIA) (VLA-4 subunit beta) (CD antigen CD29)	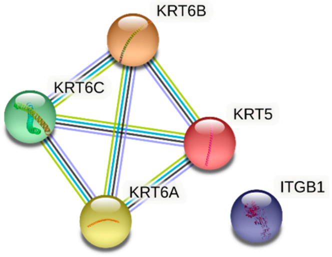	N/A

Q92896	GSLG1_HUMAN	Golgi apparatus protein 1 (CFR-1) (Cysteine-rich fibroblast growth factor receptor) (E-selectin ligand 1) (ESL-1) (Golgi sialoglycoprotein MG-160)	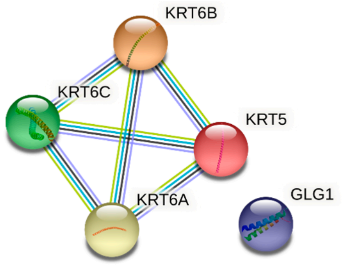	N/A
Q9NZM1	MYOF_HUMAN	Myoferlin (Fer-1-like protein 3)	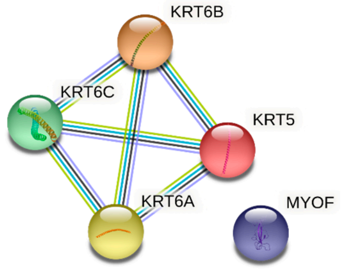	N/A
O75976	CBPD_HUMAN	Carboxypeptidase D (EC 3.4.17.22) (Metallocarboxypeptidase D) (gp180)	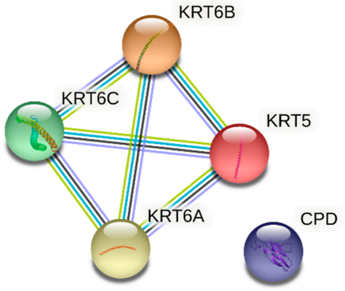	N/A
Q13740	CD166_HUMAN	CD166 antigen (Activated leukocyte cell adhesion molecule) (CD antigen CD166)	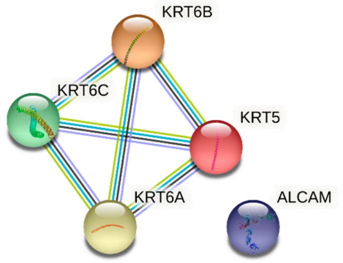	N/A
P10314	1A32_HUMAN	HLA class I histocompatibility antigen, A-32 alpha chain (MHC class I antigen A*32)	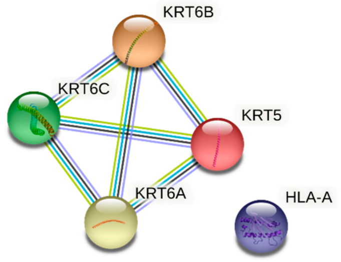	N/A
Q6UVK1	CSPG4_HUMAN	Chondroitin sulfate proteoglycan 4 (Chondroitin sulfate proteoglycan NG2) (Melanoma chondroitin sulfate proteoglycan) (Melanoma-associated chondroitin sulfate proteoglycan)	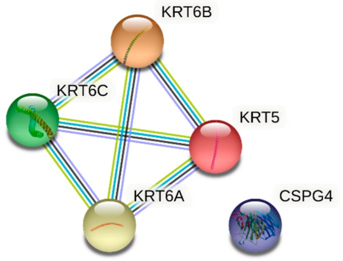	N/A

O15427	MOT4_HUMAN	Monocarboxylate transporter 4 (MCT 4) (Solute carrier family 16 member 3)	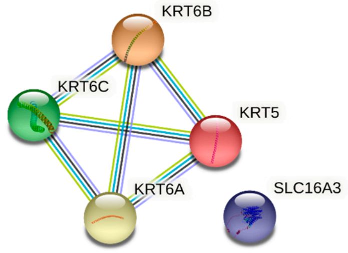	N/A
Q07065	CKAP4_HUMAN	Cytoskeleton-associated protein 4 (63-kDa cytoskeleton-linking membrane protein) (Climp-63) (p63)	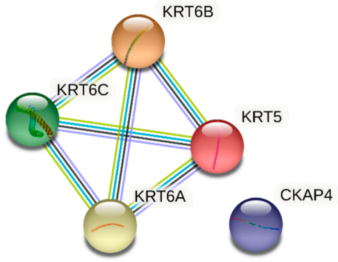	N/A
P23634	AT2B4_HUMAN	Plasma membrane calcium-transporting ATPase 4 (PMCA4) (EC 3.6.3.8) (Matrix-remodeling-associated protein 1) (Plasma membrane calcium ATPase isoform 4) (Plasma membrane calcium pump isoform 4)	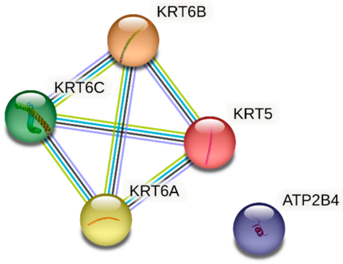	N/A
P05023	AT1A1_HUMAN	Sodium/potassium-transporting ATPase subunit alpha-1 (Na(+)/K(+) ATPase alpha-1 subunit) (EC 3.6.3.9) (Sodium pump subunit alpha-1)	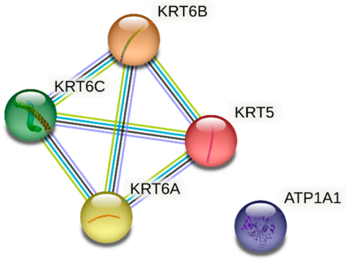	N/A
Q07954	LRP1_HUMAN	Prolow-density lipoprotein receptor-related protein 1 (LRP-1) (Alpha-2-macroglobulin receptor) (A2MR) (Apolipoprotein E receptor) (APOER) (CD antigen CD91)	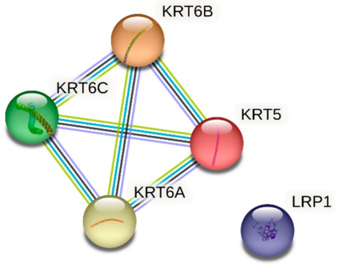	N/A
P18084	ITB5_HUMAN	Integrin beta-5	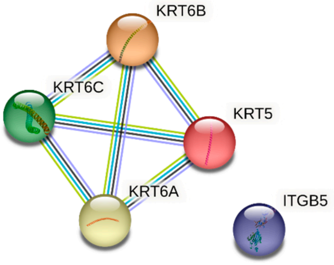	N/A
Q03135	CAV1_HUMAN	Caveolin-1	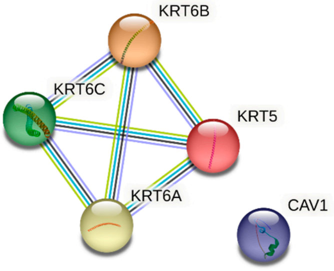	N/A

Q9UKX5	ITA11_HUMAN	Integrin alpha-11	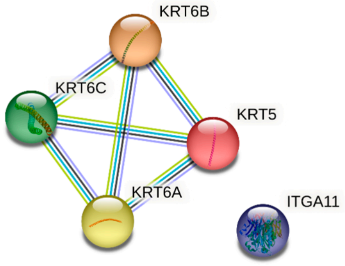	N/A
Q92544	TM9S4_HUMAN	Transmembrane 9 superfamily member 4 (Tumor cannibalism associated protein 1)	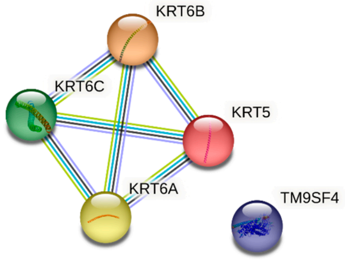	N/A
P26006	ITA3_HUMAN	Integrin alpha-3 (CD49 antigen-like family member C) (FRP-2) (Galactoprotein B3) (GAPB3) (VLA-3 subunit alpha) (CD antigen CD49c)	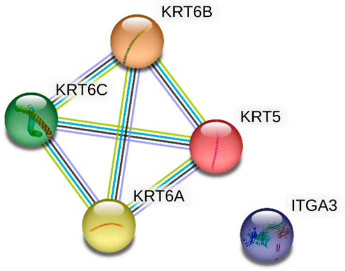	N/A
P06756	ITAV_HUMAN	Integrin alpha-V (Vitronectin receptor) (Vitronectin receptor subunit alpha) (CD antigen CD51)	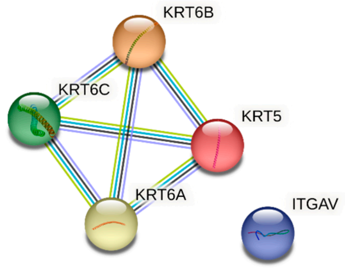	N/A
P08648	ITA5_HUMAN	Integrin alpha-5 (CD49 antigen-like family member E) (Fibronectin receptor subunit alpha) (Integrin alpha-F) (VLA-5) (CD antigen CD49e)	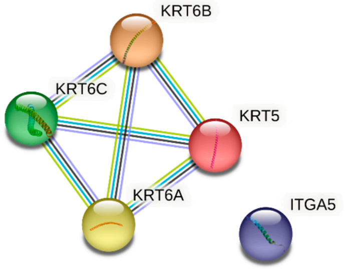	N/A
O14786	NRP1_HUMAN	Neuropilin-1 (Vascular endothelial cell growth factor 165 receptor) (CD antigen CD304)	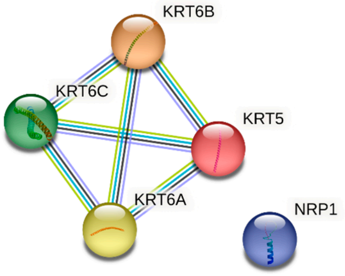	N/A

P10321	1C07_HUMAN	HLA class I histocompatibility antigen, Cw-7 alpha chain (MHC class I antigen Cw*7)	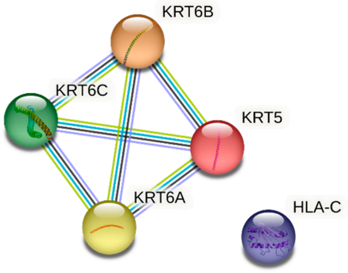	N/A
P05362	ICAM1_HUMAN	Intercellular adhesion molecule 1 (ICAM-1) (Major group rhinovirus receptor) (CD antigen CD54)	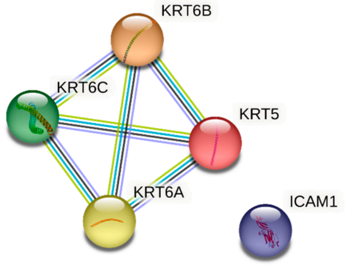	N/A
P22413	ENPP1_HUMAN	Ectonucleotide pyrophosphatase/phosphodiesterase family member 1 (E-NPP 1) (Membrane component chromosome 6 surface marker 1) (Phosphodiesterase I/nucleotide pyrophosphatase 1) (Plasma-cell membrane glycoprotein PC-1)	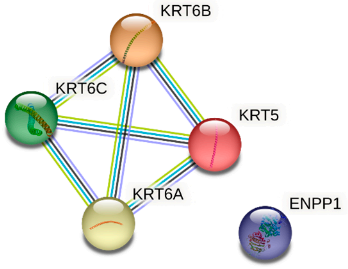	N/A
P35580	MYH10_HUMAN	Myosin-10 (Cellular myosin heavy chain, type B) (Myosin heavy chain 10) (Myosin heavy chain, non-muscle IIb) (Non-muscle myosin heavy chain B) (NMMHC-B) (Non-muscle myosin heavy chain IIb) (NMMHC II-b) (NMMHC-IIB)	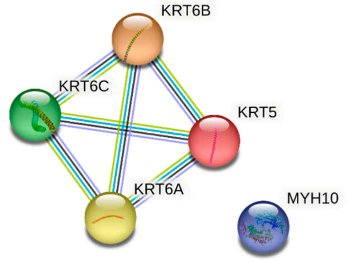	N/A
P02786	TFR1_HUMAN	Transferrin receptor protein 1 (TR) (TfR) (TfR1) (Trfr) (T9) (p90) (CD antigen CD71)	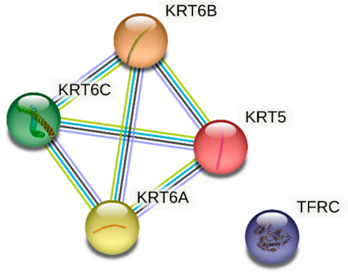	N/A

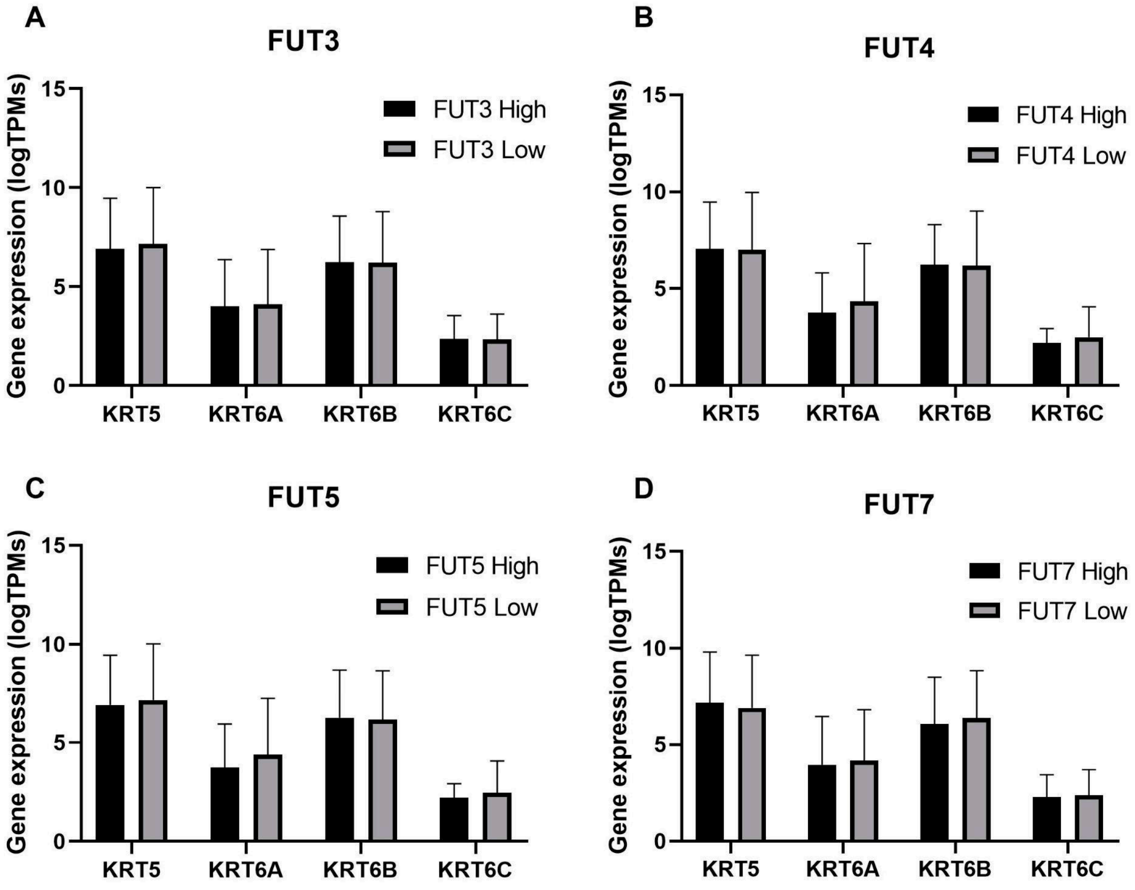

Figure S1. - CK5/6 genes expression according to the high or low expression of FUTs. TNBC tissue genetic data from the TCGA database was used to evaluate gene expression. The samples were subdivided based on the median expression of various 1,3-fucosyltransferases (*FUT3, FUT4, FUT5* and *FUT7*) and correlated with genes involved in cytokeratin production (*KRT5, -6A, -6B, -6C*).

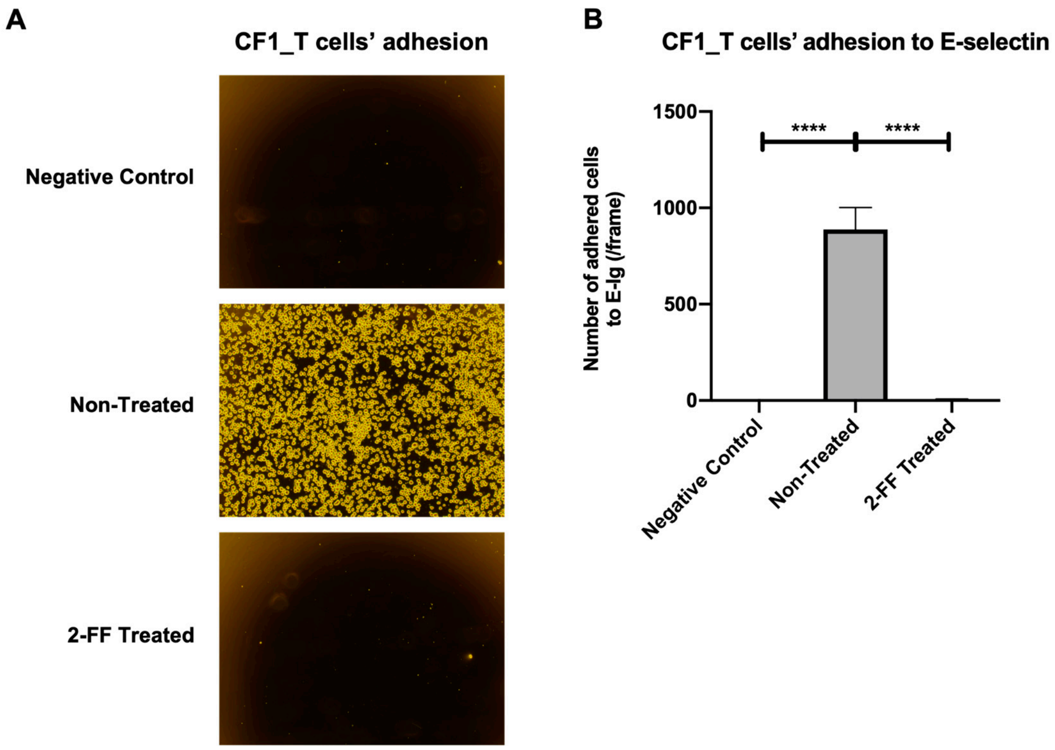

Figure S2. CF1_T cells treated with 2-FF lose the ability to bind E-selectin under flow conditions. (A) CF1_T were treated or not with 1 mM 2-FF for 5 days and resuspended in Hank's balanced salt solution (HBSS) containing 2 mM CaCl2 (HBSS-Ca). They were overlaid in glass slides spotted with E-Ig chimaera, previously blocked with 1% BSA. Their ability to bind to E-Ig chimaera was analysed under flow conditions, specifically using an orbital rotation at 80 r.p.m. for 30 min at 4 °C. The slides were immersed in HBSS-Ca to remove the non-adherent cells, and adherent cells were fixed with 3% glutaraldehyde. Assays performed in EDTA buffer were used as negative control. Adherent cells were examined under light microscopy at 100× magnification. The photomicrographic sections are a representative sample of a $n = 4$. (B) The number of adherent cells in each photomicrograph was counted using ImageJ software. [$p < 0.0001$ (****)].

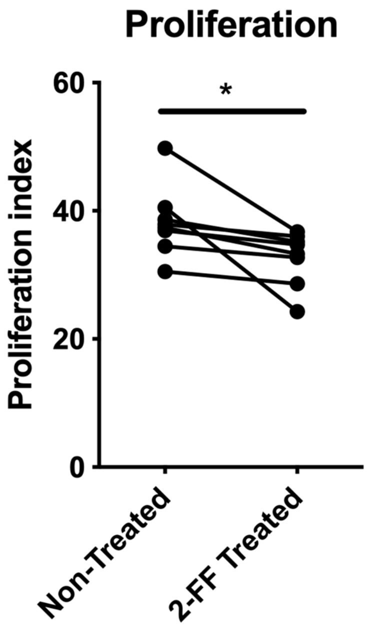

Figure S3. Proliferation index of CF1_T cells treated with 2-fluorofucose (2-FF). CF1_T cells were treated, or not, with 2-FF, for a total of 14 days. At day 5 of treatment, the cells were stained with CFSE dye and then analysed after another 9 days of treatment (total of 14 days). CFSE dilution was analysed by flow cytometry, and the proliferation index was calculated as the fold expansion of the overall culture using the MODFIT software [$n = 8$; $p < 0.05$ (*)].

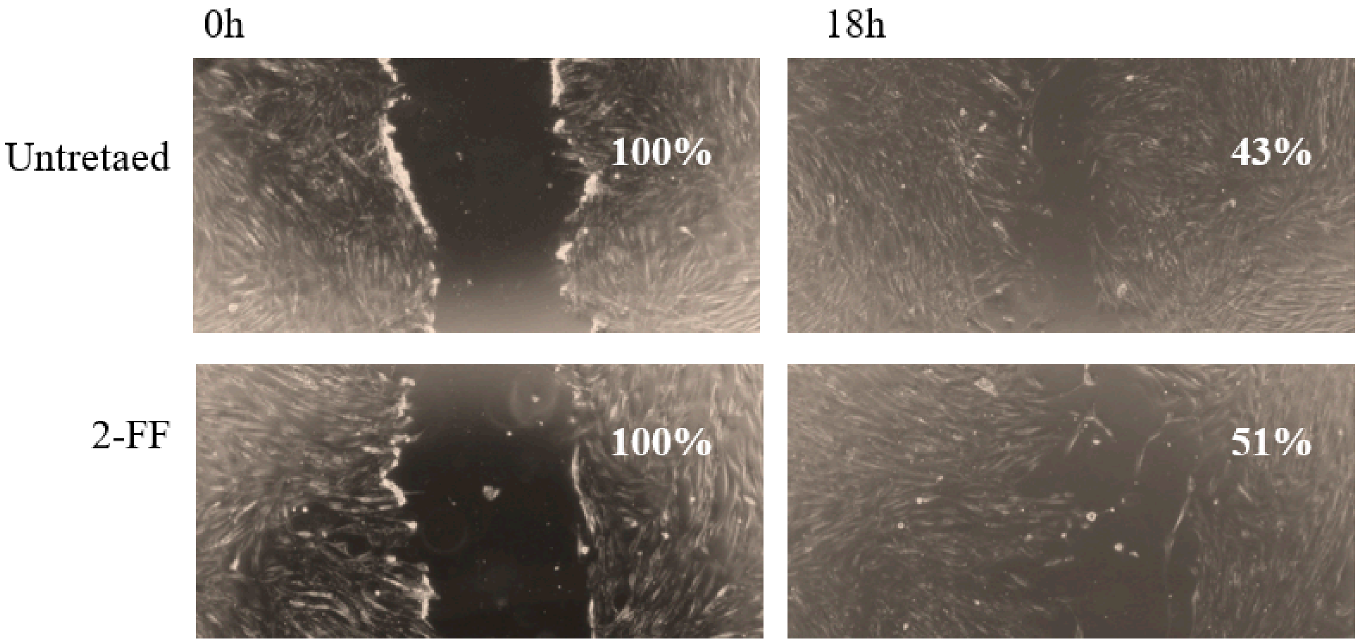

Figure S4. Migratory ability of CF1_T cell line after 2-fluorofucose (2-FF) treatment analysed by scratch wound healing assay. CF1_T cells were seeded into 12-well microplates and grown to confluence in the presence or absence of 1mM of 2-FF inhibitor. After wounding the monolayer with a pipette tip. The suspended cells and debris were washed away, and fresh medium was added. At 0 and 18 h after wounding, scratched regions were photographed with an inverted microscope equipped with a digital camera. The percentage of wounded area in the photographs is indicated in white. It was measured using ImageJ software and the percentage of the closed area was calculated using the formula: ((wounded area (18h)/wounded area (0h)) *100) – 100)). .

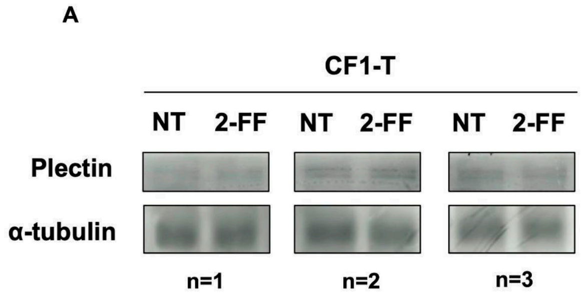

B

Lane	Plectin intensity	α-tubulin intensity	Ratio plectin/α-tubulin
#2 NT	284.28	13450.86	0.02
#2 T	1845.84	11239.07	0.16
#3 NT	4436.86	17374.32	0.25
#3 T	4297.98	20084.22	0.21
#4 NT	3930.64	16042.87	0.24
#4 T	2280.38	13091.99	0.17

Figure S5. CF1_T cell line expresses plectin. (A) CF1_T cell lines were treated with 2-FF inhibitor for 5 days and total lysate proteins were stained with $\alpha6$ integrin antibody and analysed by western blot (n = 3). α-tubulin expression was analysed as a loading control. **(B)** Densitometry readings of Western blot films regarding plectin and α-tubulin bands intensity. The ratios between plectin and α-tubulin intensities are also presented. Whole Western blot membranes are included in Figure 1 of Supporting Information.
